# Convergent Adaptations: Bitter Manioc Cultivation Systems in Fertile Anthropogenic Dark Earths and Floodplain Soils in Central Amazonia

**DOI:** 10.1371/journal.pone.0043636

**Published:** 2012-08-29

**Authors:** James Angus Fraser, Alessandro Alves-Pereira, André Braga Junqueira, Nivaldo Peroni, Charles Roland Clement

**Affiliations:** 1 Departamentos de Antropología e Historia, Universidad de los Andes, Bogotá, Cundinamarca, Colombia; 2 Department of Anthropology, University of Sussex, East Sussex, United Kingdom; 3 Post-graduate Program in Genetics and Plant Breeding, Universidade de São Paulo, Piracicaba, Estado de São Paulo, Brazil; 4 Laboratório de Evolução Aplicada, Universidade Federal do Amazonas, Manaus, Amazonas, Brazil; 5 Coordenação de Tecnologia e Inovação, Instituto Nacional de Pesquisas da Amazônia, Manaus, Amazonas, Brazil; 6 Centre for Crop Systems Analysis/Technology and Agrarian Development Group Wageningen University, Wageningen, Gelderland, The Netherlands; 7 Departamento de Ecologia e Zoologia, Universidade Federal de Santa Catarina, Florianópolis, Santa Catarina, Brazil; The Pennsylvania State University, United States of America

## Abstract

Shifting cultivation in the humid tropics is incredibly diverse, yet research tends to focus on one type: long-fallow shifting cultivation. While it is a typical adaptation to the highly-weathered nutrient-poor soils of the Amazonian *terra firme*, fertile environments in the region offer opportunities for agricultural intensification. We hypothesized that Amazonian people have developed divergent bitter manioc cultivation systems as adaptations to the properties of different soils. We compared bitter manioc cultivation in two nutrient-rich and two nutrient-poor soils, along the middle Madeira River in Central Amazonia. We interviewed 249 farmers in 6 localities, sampled their manioc fields, and carried out genetic analysis of bitter manioc landraces. While cultivation in the two richer soils at different localities was characterized by fast-maturing, low-starch manioc landraces, with shorter cropping periods and shorter fallows, the predominant manioc landraces in these soils were generally not genetically similar. Rather, predominant landraces in each of these two fertile soils have emerged from separate selective trajectories which produced landraces that converged for fast-maturing low-starch traits adapted to intensified swidden systems in fertile soils. This contrasts with the more extensive cultivation systems found in the two poorer soils at different localities, characterized by the prevalence of slow-maturing high-starch landraces, longer cropping periods and longer fallows, typical of previous studies. Farmers plant different assemblages of bitter manioc landraces in different soils and the most popular landraces were shown to exhibit significantly different yields when planted in different soils. Farmers have selected different sets of landraces with different perceived agronomic characteristics, along with different fallow lengths, as adaptations to the specific properties of each agroecological micro-environment. These findings open up new avenues for research and debate concerning the origins, evolution, history and contemporary cultivation of bitter manioc in Amazonia and beyond.

## Introduction

Shifting cultivation has been a predominant mode of traditional agriculture in the rainforests of the lowland Neotropics [1], humid Africa [2], the Indian subcontinent [3] and parts of South East Asia [4] and Oceania [5] for thousands of years. Shifting cultivation in the humid tropics is incredibly diverse [6], yet both empirical studies and theoretical discussions tend to focus on one type: long-fallow shifting cultivation (LFSC). LFSC is an *extensive* form of crop production, which entails a relatively short cropping period of 1–3 years, followed by a longer fallow period (normally 10–20 years), with land cleared by slashing and burning fallow vegetation (or sometimes mature forest) [7]. The long fallow period is necessary because soils of the *terra firme* (non-flooded upland plateaus) in the tropical world tend to be highly-weathered infertile Oxisols and Ultisols (US classification system). Hence, this form of land-use is spatially (fields must shift frequently) and temporally (fields must be fallowed for years before they can be cultivated again) *extensive*. Extensive systems are widely held to be well adapted to such soils because a nutrient flush provided by the burning of secondary or mature forest is used for a single cropping period, before swidden fields are left to fallow in order to restore fertility via the growth of secondary forest [8]. It has long been assumed that any intensification of LFSC (i.e., a reduction of fallow periods) in the humid tropics will cause crop yields to decline, as infertile Oxisols and Ultisols are not capable of withstanding a sustained reduction of fallow periods, leading to the eventual collapse of the system. These assumptions about the prevalence and precariousness of extensive shifting cultivation have influenced theories of cultural development in Amazonia, where Steward [9] and Meggers [10] held that the small scattered Amerindian settlements practicing LFSC found in the interfluvial *terra firme* today represent an optimal adaptation to a low productivity environment. While their theories have long since gone out of fashion [11], studies of the cultivation of the Amazonian staple bitter manioc (*Manihot esculenta* Crantz) continue to focus almost exclusively on LFSC practiced by current Native Amazonian populations [12] and other traditional populations [13] in the poor soils of marginal interfluvial *terra firme* environments.

In Amazonia, however, the most populated areas – both today and in the late pre-Columbian period – are along major white-water rivers, such as the Solimões, Amazon and Madeira, all of which have nutrient-rich floodplain soils used for agriculture and adjacent paleo-floodplains that are somewhat more nutrient-rich than the typical Oxisols and Ultisols [14], as well as areas of Anthropogenic Dark Earths [15]. These regions are among the most suitable for human habitation and population growth, since they are characterized by an abundance of fish as well as fertile soils. Students of pre-Columbian Amazonia have long emphasized the agricultural potential of these regions. Lathrap [16] argued that the productivity of the white-water floodplains underwrote the growth of large and dense human settlements, sparking competition and warfare between groups for these rich but spatially and temporally limited areas. However, although the flood pulse provides a yearly flush of nutrients, extreme floods once every decade or so can destroy crops, settlements and even the high floodplain itself, which makes these environments relatively unpredictable. Denevan [17] argued that agriculture began as multi-cropping in floodplain environments and developed later in forested uplands as intensive multi-cropping systems with short fallow. For him, long fallow, extensive cultivation is a late development associated with the introduction of efficient forest clearance tools, especially metal axes and machetes.

Denevan recognized that large scale settlements could not have been supported exclusively by seasonal floodplain cultivation, given the riskiness of producing food in areas subjected to floods. He proposed that settlements would preferentially be located on bluffs along the river margins, allowing the exploitation of both the fertile floodplain zones and the *terra firme* areas that were safe from flooding but located on less fertile soils [17], [18]. This occupation pattern would ultimately result in the transformation of upland soils: many bluffs in central Amazonia feature fertile anthropogenic soils known as Anthropogenic Dark Earths (ADE) [19]–[21]. These soils are associated with Native Amazonian settlements of the late pre-Columbian period (2000–500 years before present) [22], [23], and are most abundant and largest in whitewater regions, because these are where the largest populations were located in pre-Columbian times [24]. Amazonian ADE form through human inputs of organic and inorganic matter [e.g., biomass wastes, manure, bones, ash, charcoal and ceramics] [15]. Amazonian ADE are enduringly fertile in part due to their historical enrichment in highly stable black carbon, which has a half-life of 1000 years [25]. ADE exhibit much higher levels of chemical elements essential for plant growth, such as phosphorous, calcium, magnesium, zinc and manganese, than the weathered and infertile soils in which they were formed. ADE sites exhibit a highly fertile ‘core area’, which grades into more subtly modified soils, with a continuum of fertility between them and surrounding soils [26], [27]. It is likely that ADE were appreciated for plant cultivation by pre-Columbian Native Amazonian peoples, just as they are by Amazonian people today [28].

These historical arguments point to the possibility that local people may also intensify shifting cultivation when circumstances permit today. Fertile soils, such as ADE and floodplain soils, provide farmers with the opportunity to intensify shifting cultivation, increasing both crop productivity and frequency of cultivation [29], raising the carrying capacity of the landscape [30]. Therefore, the study of swidden systems on fertile soils allows us to examine the extent to which fallow lengths, bitter manioc landraces, and other aspects of swidden systems vary on different soils. While numerous studies have addressed many dimensions of shifting cultivation, few have compared swidden systems on different soils and in different ecological contexts within a circumscribed geographical area [31].

### The Amazonian Staple: Bitter Manioc

Manioc landraces are classified as either bitter or sweet depending on cyanogenic glucoside (CG) content. In Central Amazonia, bitter manioc is the staple crop today, whilst sweet manioc is only of secondary importance [32]. Sweet manioc has low CG content in their tuberous roots (<100 ppm fresh weight), while bitter manioc has larger amounts of cyanogenic glycosides (>100 ppm fresh weight) [33]. There are an estimated 7000 landraces of manioc worldwide [34], but this is surely an underestimate given farmers' continual selection and propagation of new landraces. Differences in the color, form and size of leaves and stems, in the size, number, color and cyanide content of roots, and in the rate of growth of tuberous roots are determined by genetic differences, although the environmental conditions in which plants grow also affect manioc phenotypes, including CG content [33]. Farmers comprehend a “landrace” as a set of individuals sharing particular morphological characteristics that differentiate them from other landraces; they also distinguish them by giving them a particular name [35], [36]. These morphological characteristics are shaped by genotype-environment interactions that can result in different phenotypic expressions of the same genotype, and create morphotypes that are identified as distinct landraces [37]. Landraces are the result of generations of farmer selection in local environments, and are therefore well adapted to local growing conditions, which has been demonstrated in South America [13], [38], [39], in humid Africa [40] and Asia [41]. Several students of manioc have asserted that bitter manioc does not yield well in fertile soils [42]–[44], perhaps because it has been so thoroughly studied on nutrient-poor soils. However, about 30% of Central Amazonian manioc, both bitter and sweet, is produced in the floodplain [45]. It was observed that on highly fertile soils manioc plants tend to invest more in aboveground biomass than in the tubers [29], [43]. However, there are bitter manioc landraces that yield well in the floodplain and in ADE, possibly even better than in Oxisols and Ultisols [46].

Manioc is vegetatively propagated via stem cuttings, which grow into plants that are genetically identical to the mature plants from which they were cut. However, manioc retains its ability to reproduce sexually and produces seeds that lie dormant in fallow vegetation [47]. When fallows are cleared for cultivation, seeds are stimulated to germinate by increasing temperatures caused by both soil exposure and the heat of the burn phase in swidden-fallow systems [48], and seedlings appear [49]. When cuttings from seedlings are incorporated into a landrace, its genetic diversity is increased because it becomes a polyclonal landrace [36], [48], [50]–[52]. This increased genetic diversity provides the raw material for adaptation to new conditions, such as ADE and the floodplain, as different clones in the landrace have different possibilities for adaptation to different agro-ecosystems. The extent to which volunteer seedlings are incorporated is variable in modern bitter manioc cultivation in the Neotropics. Studies in Guyana [53], French Guiana [54] and the Atlantic Forest in Brazil [55] found a relatively high level of incorporation. However, Stocker found only a small amount of seedling incorporation amongst farmers in Pará, in eastern Brazilian Amazonia ([56]:162–63). Nonetheless, this is a recurring pattern in many traditional farming systems in Amazonia and beyond [13].

This article examines the hypothesis that contemporary Amazonian people have developed divergent bitter manioc cultivation systems as adaptations to the properties of different soils in the landscapes that they inhabit. We test this hypothesis by comparing swidden systems in fertile (ADE and floodplain) and infertile (Oxisols and Ultisols) soils with respect to their assemblages of manioc landraces, the performance (from the farmer's point of view) and productivity of the most common landraces in different soils, along with the genetic relationships between the most common landraces, and length of fallow periods. Hence, we will examine both the adaptations of manioc landraces and of swidden-fallow systems. In earlier publications [46], [57], [58], we hypothesized, based on initial interpretations of ethnobotanical data, that “weak” bitter manioc landraces grown in ADE originated in the floodplain, given farmers' knowledge and similar adaptations (fast maturation and low starch content).

## Results and Discussion

### Farmer theory and practice

**Figure 1:**
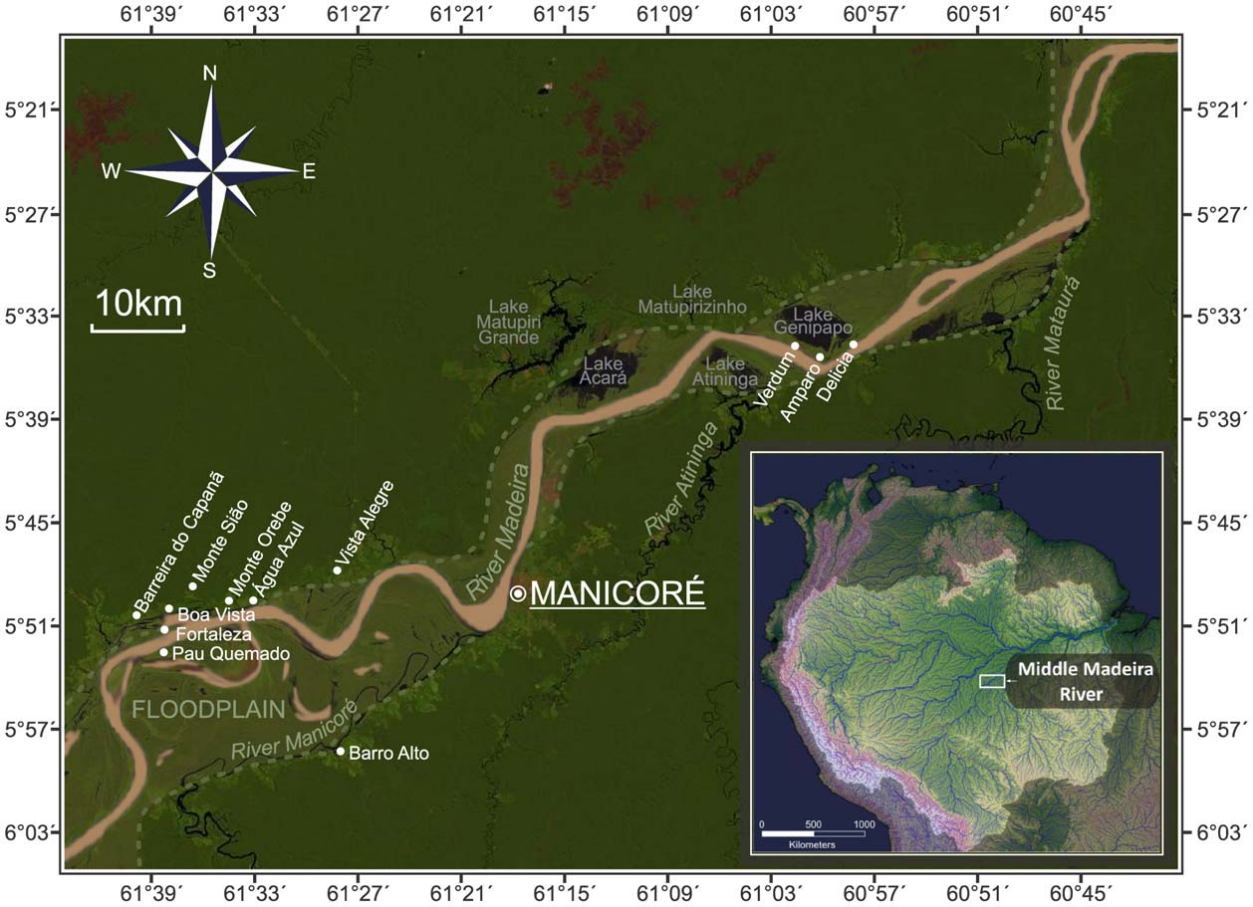
Map of the middle Madeira River region, Amazonas State, Brazil, showing communities where research was carried out. The inset map shows the location of the middle Madeira River in Northern South America. Map by Victoria Frausin.

In the preliminary stage of research we conducted multi-sited participant observation in numerous communities of the study region (Figure 1). Subsequent quantitative work was driven by hypotheses generated during this stage. While qualitative data have been presented at length elsewhere [57], here we summarize major findings and their interplay with quantitative data. We found that, contrary to what was expected from the literature [29], [31], [43], [59]–[61], bitter manioc was widely cultivated in ADE. More specifically, farmers stated that not only did they *i*) perceive that certain landraces yield better in certain soils (the basis for our *pri* index and yield observations, see below), but that this informed their *ii*) selection of different landraces for planting in different soils (the basis for the *la* index), and *iii*) that fertile soils (ADE/floodplain) could be farmed with much shorter fallow periods than infertile soils (the basis for our fallow length data).

Most intriguingly, however, was the finding that farmers expressed these perceptions and behaviors in the form of a local theory of strength and weakness (the categories strong and weak are present in traditional agriculture in other areas of the world ([62]:148)). Along the middle Madeira River, the categories weak and strong express the suitability of different landraces for planting in different soil-successional scenarios. Briefly, weak landraces are fast yielding (5 months–1 year), but rot if left too long in the ground, and produce less starch than stronger landraces. Strong landraces, on the other hand, are slow yielding (1–3 years), but produce more starch than weaker landraces. Farmers claim that weak landraces are best suited to planting in “weak” land (land with young fallow), whilst strong landraces are said to be suited to “strong” land (land with older fallow). Elsewhere, we reported that landraces described as weak were planted more frequently in ADE and the floodplain, whilst those described as strong were planted more frequently in Oxisols and Ultisols ([63]:400). The specifics of this local theory have been elaborated at length [46], [57], [58], and here we would just emphasize two points. This local theory would appear to provide evidence of the cognitive aspects of adaptation – local people have theorized the emergent properties of their adaptive knowledge and practices – and express these using the simple yet powerful metaphors of strength and weakness. Secondly, this theory is not limited to the middle Madeira; an independent study recently found an identical theory of weak and strong manioc amongst floodplain and *terra firme* manioc farmers in and around the Brazilian Sustainable Development Reserves of Mamirauá and Amanã, close to the town of Tefé on the middle Solimões River [64]. The fact that the same local theory exists in two localities hundreds of kilometers apart suggests that this theory is likely to inform adaptive aspects of manioc farming in various locations throughout Amazonia.

### Diversity of manioc landraces

A total of 50 landraces were found, with 29 in ADE, 20 in Oxisols, 20 in Ultisols and 23 in the floodplain. Most of the landraces cultivated on the *terra firme* are planted in more than one type of soil: among the 29 landraces cultivated in ADE, 18 (62%) are also planted in Ultisols and 19 (65.5%) in Oxisols. Ultisols and Oxisols shared 75% (15) of their landraces. On the other hand, most of the landraces that occurred in the floodplains (19, or 82.6%) only occurred in this environment, with only four landraces shared with other types of soil (one shared with Ultisols and ADE, and three that occurred in all four types of soil). The number of landraces cultivated in each village varied between 5 (Verdum) and 21 (Vista Alegre), and tended to be lower in floodplain villages (N = 5; mean ± standard deviation: 8.2±3.1) than villages located on the *terra firme* (N = 4; 15.7±5.0), principally because villages on the *terra firme* have access to more than one type of soil (and, in the case of Água Azul, even cultivate in the floodplain). The average number of landraces cultivated in each manioc field was similar among the different types of soil (ADE: 2.8±1.9; Oxisols: 3.4±1.5; Ultisols; 3.2±2.0; Floodplain: 3.1±1.3) (Dataset S1).

Along the Upper Negro River, communities commonly have 60 to 89 landraces, somewhat higher when compared to other local communities in Amazonia and in the Atlantic Forest in Brazil (53–58; [13]). The diversity of bitter manioc landraces on the *terra firme* along the middle Madeira is quite low, although higher than what Grenand reported along the Cuieiras River near Manaus (six) [65]. Perhaps this is due to a greater market orientation in the Madeira River communities, and higher population pressure. By contrast, the number of landraces we registered on the floodplain is much higher than reports for Careiro Island (three; [65]) at the confluence of the Negro and Solimões Rivers, and for communities between Santarém and Óbidos (maximum of four), along the middle Amazon River [66], although Pereira [67] reported 43 bitter manioc landraces at the confluence of the Solimões and Japurá Rivers. The generally low numbers in the literature most likely reflect the lack of attention to manioc in the floodplain compared to the *terra firme*, as pointed out by Denevan [68] for studies of agriculture in the floodplain in general. Various studies mention fast maturing manioc in the floodplain, without reporting the number of landraces [69]–[71].

### Landrace composition of manioc fields in different soils

**Figure 2:**
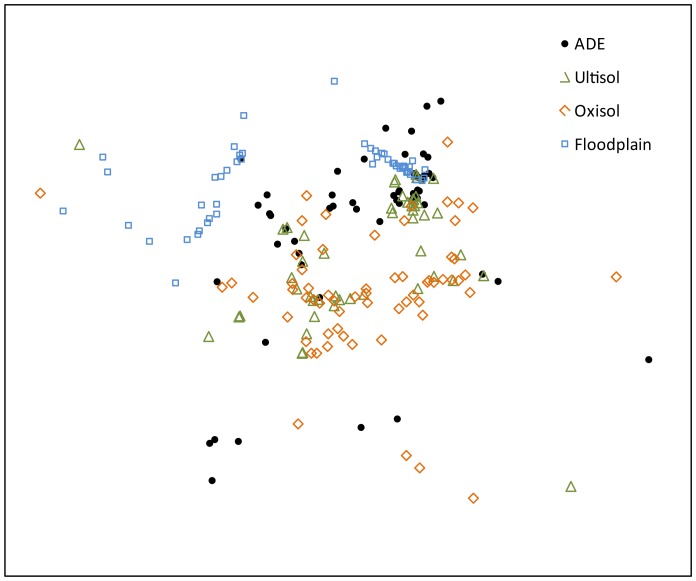
Non-metric multidimensional scaling (NMDS) showing the composition of bitter manioc landraces sampled in fields on Ultisols, Oxisols, Anthropogenic Dark Earths (ADE) and on floodplains along the middle Madeira River (percentage of explanation of the bidimensional model: 35.8%). Each point represents a bitter manioc field, and its position in the graph is a bidimensional representation of the Bray-Curtis dissimilarity between the fields.

The composition of manioc landraces in fields on different types of soil is significantly different, which is shown both in the general model (NPMANOVA; F = 16.46, p = 0.01) and also in all possible pairwise comparisons (six) between the four different soil types (all with p values lower than 0.05, corrected for multiple comparisons) (Figure 2). In general, manioc fields in Oxisols and Ultisols cluster together, while in Floodplains they form an almost completely separate group, with fields in ADE occupying an intermediate area in the ordination space (Figure 2). Also, fields in Oxisols and Ultisols are more homogeneous, while in Floodplains and ADE they are more heterogeneous, especially in the latter case. The areas of overlap and the dispersion of some points through the figure indicate that the composition of manioc landraces is very heterogeneous and highlights the practice of landrace exchange between soils. Within-field heterogeneity is commonly reported [12], [13], [38], [72], as is exchange among farmers [35], [36], [38]–[40], [48], [73], but exchange among farmers on different soil types has not been reported previously, perhaps because the heterogeneity of soils has been less carefully studied than that of bitter manioc landraces.

### Farmer perceptions of landrace performance

**Figure 3:**
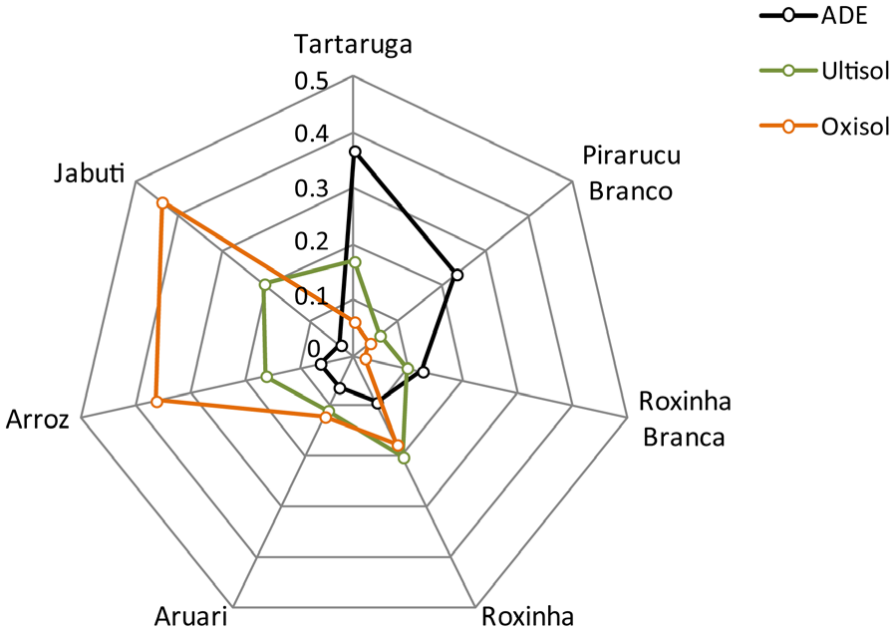
Farmer perceptions of performance for the six most frequent manioc landraces cultivated in Oxisols, Ultisols and Anthropogenic Dark Earths (ADE) along the middle Madeira River. Numbers in the axis indicate the Performance-Rank Index (*pri*) of each landrace in each type of soil, calculated based on the perception of 162 farmers interviewed on the Middle Madeira. Dots indicate the value of *pri* for each variety in the three types of soil.

A Performance Ranking Index (*pri*) for each landrace in each type of soil on the *terra firme* was calculated. Most of the landraces mentioned by the farmers in the interviews in all three soils on the *terra firme* (ADE, Oxisol and Ultisol) have a very low *pri* score, either because they are rarely cited in that specific soil or because farmers do not rank them among the best performers in that soil (Figure 3) (Dataset S1). *Roxinha*, *Tartaruga*, *Arroz* and *Aruari* are among the five landraces with higher *pri* in all three soils. However, while the landraces *Jabuti* and *Arroz* are considered the best performers in Oxisols and Ultisols, in ADE this place is occupied by *Tartaruga*, *Pirarucu Branco* and *Roxinha Branca* (Figure 3).

There were strong positive correlations between the area occupied by each landrace in the fields (*la*) and their performance (*pri*) in the soils (Pearson's Correlation - Ultisol: ρ = 0.901; Oxisol: ρ = 0.912; ADE: ρ = 0.898). This high degree of correlation between *pri* and *la* supports the notion that farmers' planting behavior is shaped by their perceptions of the performance of different landraces in different types of soil. While this pattern is common and has been mentioned elsewhere (e.g. [74]:50), the correlations between this pair of indices permits a quantitative demonstration of this behavior.

### Farmer estimates of manioc yields

Our farmer yield observations reveal that there are significant differences between yields in five of the six most popular landraces when planted in different soils along the middle Madeira River (Table 1). These findings support our inference of a high degree of adaptation in bitter manioc systems found on different soils, since landraces that perform better in particular soils tend to be more predominant in those soils, as farmers respond positively to good yields by planting more of specific landraces. This is reflected in the *la* and *pri* indices: *Jabuti* and *Roxinha* are significantly more predominant in Oxisols and Ultisols than in ADE, while *Tartaruga* and *Pirarucu Branco* are more predominant in ADE than in Oxisols or Ultisols.

**Table 1:** Farmer-recorded production of 50 kg sacks of manioc flour that would be obtained from approximately 0.5 ha of swidden plot at various localities along the Middle Madeira River, Central Amazonia, Brazil, in 2007–2008.

Manioc landrace	ADE		Oxisol/Ultisol		Floodplain		ANOVA	
	n	(avg ± SD)	n	(avg ± SD)	n	(avg ± SD)	F	p
Jabuti	5	24.2±5.1	6	44.2±8.6			20.6	0.001
Arroz	4	31±2.6	4	30.5±4.2			0.04	ns
Tartaruga	6	44.4±6.1	7	26.7±3.5			43.0	<0.001
Roxinha	5	25.2±3.11	7	40.1±4.3			43.5	<0.001
Pirarucu Branco	4	53.2±7.0^a^	4	25.7±4.6^b^	5	55±11.2^a^	15.8	0.001
Pirarucu Amarelo	4	66.7±24.9^a^	4	30.5±4.4^b^	4	70.5±11.3^a^	7.6	0.012

Superscript characters indicate significant differences in pairwise comparisons using Tukey's HSD test (p<0.05).

### Fallow length and the intensification of swidden systems

Intensification in swidden-fallow systems refers, among other factors, to a shortening of fallow length [75]. Intensification on infertile soils can result in exhaustion of fertility and breakdown of the system. Fertile soils provide opportunities for sustainable reduction of fallow lengths. The average fallow length for 55 fields on Ultisols was 13.2±8.1 years and for 64 Oxisol fields the average length was 21.3±15.4 years, while for 71 ADE fields it was 6.5±6.8 years and for 59 floodplain fields it was 1.6±4.0. There were significant differences between fallow lengths in different soils (ANOVA, F = 44.51, p<0.001, while Tukey's post hoc test revealed significant differences between all possible pairwise comparisons except ADE vs Floodplain). It is important to emphasize that local factors, such as population density, also play important roles in shaping fallow lengths, and these are discussed at length for each locality in the Supporting Information, along with other factors (Text S1).

### Generation, selection and exchange of landrace diversity

Local farmers identify and exchange genetic diversity of bitter manioc landraces [38], [72]. Along the middle Madeira River, we observed that certain people that we categorized as “key individuals” play an important role in the exchange of new landraces originating from seedlings, conservation of existing landraces and knowledge associated with them. Key individuals were identified as being the individuals who most informants mentioned during open interviews as being responsible for introducing new landraces and keeping a stock of all landraces present in each community. Interviews with these key individuals revealed that they consciously recognize new phenotypes and are always intentionally experimenting with new landraces from seedlings and with clonal material from their kin in other communities [13]. These key individuals perform a vital role in the identification and distribution of new manioc genetic diversity, because they try out new landraces in local micro-environments and, if they prove to be exceptional, are responsible for their distribution within communities, and to kin in other communities. The fact that each locality only has a few key individuals points to the critical role that they play in the circulation of exceptional landraces throughout the region, but some new genotypes can circulate unconsciously mixed with other individuals that are morphologically similar.

At the six localities examined in this study, both the incorporation of seedlings into pre-existing landraces and the creation of new landraces from seedlings are relatively common practices. Farmers recognize that seedlings often appear in the newly burnt fields *before* cuttings have been planted and are morphologically different from the planted landraces, as they tend to grow taller and only have a single tuberous root growing straight downwards. Most farmers (53–66% across communities) interviewed simply ignore or weed out the seedlings, while others (11–32%, including, but not restricted to key individuals) take cuttings from mature seedlings and plant them separately (Table 2). When mature, these volunteers are either: i) incorporated into an existing landrace, e.g., *Jabuti*; ii) incorporated as a sub-landrace of an existing landrace (such as *Jabuti*-*Preto*); iii) named as a combination of two landraces (such as *Jabuti*-*Arroz*); or iv) established as a new landrace with a different name.

**Table 2:** Number of informants, both total and key individuals for manioc management, number of landraces cultivated, and the ways that farmers manage seedlings at six communities along the middle Madeira River, municipality of Manicoré, Amazonas, Brazil.

	Informants		Number of landraces	Seedling Management		
	Total	Key		Intentionally incorporate	Incorporate at random	Remove
Barro Alto	37	3	12	0.32	0.14	0.54
Barreira do Capanã/Boa Vista	29	6	19	0.17	0.14	0.66
Água Azul	13	4	9	0.23	0.31	0.46
Vista Alegre	11	4	21	0.27	0.09	0.55
Água Azul floodplain	9	3	13	0.11	0.33	0.55
Genipapo floodplain	15	4	14	0.26	0.20	0.53

All the landraces analyzed with microsatellite markers had observed heterozygosities (*H_O_*) higher than expected heterozygosities (Table 3), which appears to be related to both the incorporation of seedlings and the selection of heterozygous individuals [76]. For example, the landrace *Arroz* from ADE at Barreira do Capanã had an observed heterozygosity of 0.495 and 5 different multi-locus genotypes (MLGs) in the 20 individuals examined. The landraces *Jabuti* from Oxisols and *Tartaruga* from floodplain had 7 and 6 different MLGs, and *H_O_* of 0.567 and 0.707, respectively. The number of MLGs reflects either the incorporation of seedlings or the unintentional “mixing” of landraces (which may happen, for example, when they are morphologically similar), both of which contribute to increasing heterozygosity. Incorporation of seedlings into recognized landraces increases intra-landrace heterogeneity, because farmers tend to select the largest and healthiest volunteers, which tend to be the most heterozygous [53], [54]. These practices are critical in maintaining and amplifying the genetic diversity of manioc landraces, and in adapting landraces to new environmental conditions, such as different soils.

**Table 3:** Indices of intra-varietal genetic diversity [Observed (*H_O_*) and expected (*H_E_*) heterozygosities, and number of multi-locus genotypes (No. MLGs)] for five bitter manioc landraces cultivated in different soil types in the middle Madeira River region, based on variation detected with 10 microsatellite markers.

Landrace	Soil	Locality	*H_O_*	*H_E_*	No. MLGs	MLGs
*Pirarucu Branco*	ADE	Barreira do Capanã	0.495	0.250	2	**A**, g
	FP	Pau Queimado	0.505	0.255	2	**B**, h
*Tartaruga*	ADE	Barro Alto	0.503	0.268	2	**B**,F
	OX	Barro Alto	0.507	0.276	4	**B**, F, i, j
	FP	Verdum	0.707	0.361	5	**C**, k, l, m, n
*Arroz*	ADE	Barreira do Capanã	0.495	0.460	5	A, **D**, o, p, q
	OX	Água Azul	0.503	0.257	2	**D**, r
*Jabuti*	OX	Barreira do Capanã	0.567	0.445	6	D, **E**, s, t, u, v
*Pirarucu Amarelo*	ADE	Água Azul	0.607	0.306	2	**F**, x
	FP	Água Azul	0.607	0.307	1	**F**

Capital letters indicate the MLGs that were present in more than one landrace. Boldfaced letters indicate the most common MLG for a given landrace, while small letters indicate MLGs present at low frequencies.

**Figure 4:**
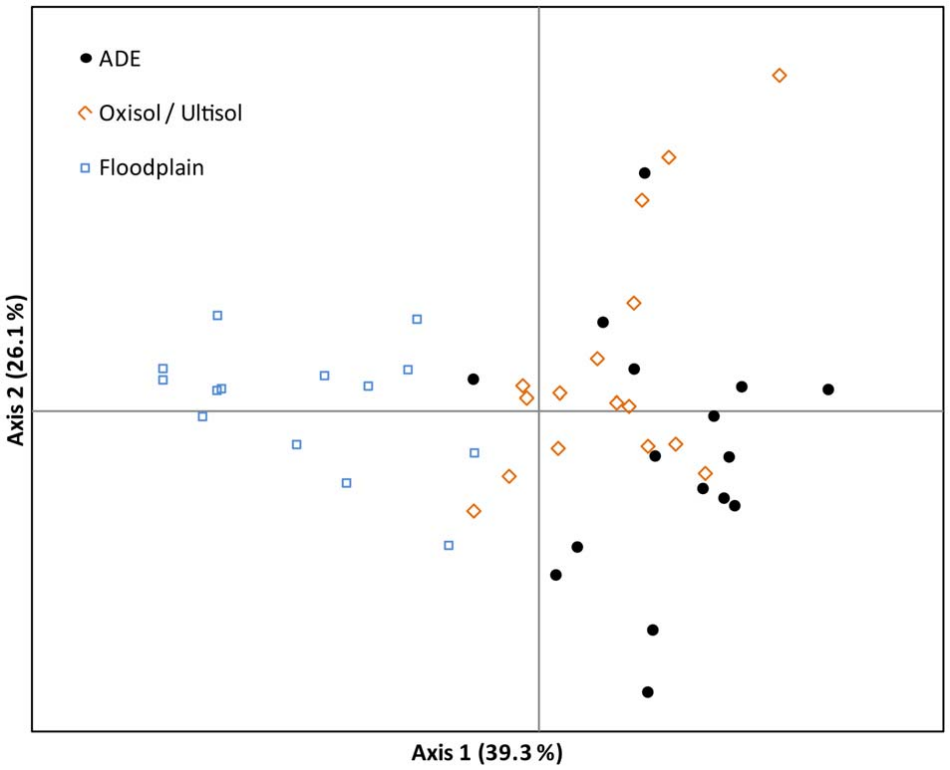
Principal Coordinates Analysis based on diversity revealed by 10 microsatellites markers showing the dispersion of 48 swiddens from three soil types (17 in ADE soils, 14 in floodplain soils and 17 in Oxisols/Utilsols) in six communities along the middle Madeira River. The two coordinates together explain 65.4% of the variation in the matrix.

**Figure 5:**
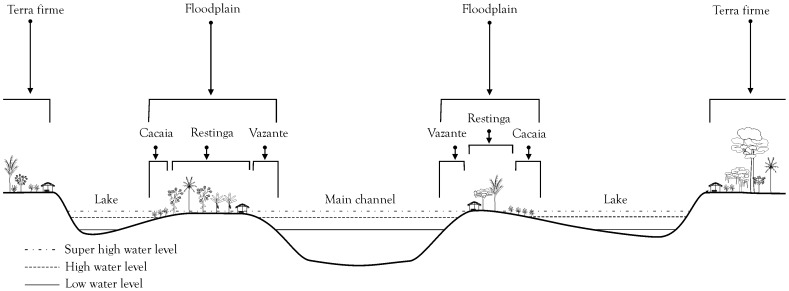
Schematic representation of floodplain zones relative to the main channel, lakes and *terra firme* (non-flooded upland plateaus). *Vazante* is the local term on the middle Madeira for the banks of the main channel, *Restinga* for the high levee floodplain and *Cacaia* for the back-swamp area. Drawn by Victoria Frausin.

Along the middle Madeira River the performance in different soils is a major factor in varietal selection by farmers (Figure 4). The selection of distinct traits in different environments where the landraces are cultivated may be correlated to the genetic differentiation found among the bitter manioc landraces grown in different soil types (Figure 5; [77]). Evidence of genetic divergence among landraces cultivated in floodplain from those cultivated in ADE and infertile soils was also found when analyzing intra-varietal genetic diversity. In all examples the landraces grown in ADE and infertile soils had the same most common MLGs. On the other hand, landraces grown in the floodplain, except *Pirarucu Amarelo*, had distinct MLGs from those with the same landrace name but grown in ADE or infertile soils. Additionally, in the floodplain some farmers observed that volunteers occur more frequently in higher areas of the floodplain. These areas are not flooded every year and the seeds have time to be acted upon by agro-ecological management. The planting of manioc in different zones, the flood regime, farmer perceptions and the combination of landraces they select drive the generation of new landraces in the floodplain. This is consistent with the somewhat higher number of landraces from the floodplain observed in this study, and may be related to the genetic differentiation among the landraces grown in the floodplain from those grown in upland soils (Figure 4).

### Farmer selection for agronomic performance? Comparative perspectives

Our findings call for re-evaluation of the widespread notion that farmer identification and selection of manioc landraces is weakly associated with agronomic performance. This notion is based on Boster's research on the perceptual distinctiveness of manioc amongst the Aguaruna Jivaro of the Peruvian Amazon. Boster concluded that “there are significant differences between the [manioc] cultivars in their responses to environmental factors…”, but “the Aguaruna are generally unaware of or unconcerned with these differences” ([72]:40). Boster's own garden experiments did, however, show that “some cultivars grow well in sandy alluvial soils on river islands and do poorly on other sites, while other cultivars yield about the same regardless of soil conditions.” Boster also noted that the Cashinahua, another Native Amazonian group that resides in Peru and Brazil, do appear to select particular landraces for planting in certain kinds of soil, according to their well-known ethnographer, Ken Kensinger [78]. The Aguaruna Jivaro reside in hill country, where there is certainly a lot of soil variation and there will be some high quality soils, and the Cashinauhua live along the fertile whitewater Juruá River (Tarauacá, Jordão, Breu, Muru, Envira) and in the Purus basin (Upper Purus and its affluent the Curanja) [79], [80]. Each of these areas offers a variety of very different soils to the manioc farmer. However, even in the Upper Negro Region, an area notorious for infertile soils, according to Wilson [81], there appears to be non-random planting of sweet manioc in more fertile soils by Tukanoan manioc farmers. Heckler and Zent, on the other hand, describing research amongst the Piaroa in Venezuela, found that “specific genotype-micro-condition interactions were demonstrably not a significant factor in determining planting patterns” ([82]:684). Similarly, Salick and colleagues, reporting on a study amongst the Amuesha in Peruvian Amazonia, found that “soil seemed to have little predictive power for cassava phenotype occurrence.” ([38]:11).

The findings of Boster, Heckler and Zent, and Salick et al. explain the persistence of the notion that farmer identification and selection of manioc landraces is weakly associated with agronomic performance. Our findings, however, when combined with those of Kensinger and Wilson, support the conclusion that the way in which *at least some* Amazonian farmers consciously identify and select landraces *is* shaped by agronomic performance. Indeed, the perceptual weak-strong theory of manioc detailed above and elsewhere appears to be built upon and indeed express in schematic/metaphoric form local knowledge of selection for agronomic performance. This raises questions regarding the distribution of practices of selection for agronomic performance, and which factors (such as the presence/absence of fertile soils, indigenous/non-indigenous farmers etc.) contribute to the presence of these practices and associated knowledge in different parts of Amazonia. We conjecture that selection for agronomic performance would be more likely to emerge amongst people inhabiting areas with greater heterogeneity of soil types.

## Conclusions

We examined manioc cultivation in four different soil types at six localities, and found that farmers plant different assemblages of bitter manioc landraces in different soils and that shorter fallow lengths were strongly associated with more fertile soils. Most popular landraces were shown to exhibit significantly different yields when planted in different soils. From this we can infer that farmers have selected different sets of landraces with different perceived agronomic characteristics, along with different fallow lengths, as adaptations to the specific properties of each agroecological micro-environment. On the *terra firme*, intentional selection of certain landraces for cultivation in certain soils was demonstrated by the close relationship between *Performance Ranking Index* and *Landrace Area* for landraces in different soil types. These findings suggest that the local theory revolving around metaphors of strength and weakness may represent a cognitive manifestation of adaptive farming behavior: a way to express in simple terms local knowledge of the relationship between different sets of bitter manioc landrace traits and soil properties and fallow stages. Although landraces grown in ADE and the floodplain share similar phenotypical characteristics, specifically fast maturation and low starch content, and are both described as “weak” by farmers, these groups of landraces were not genetically more related to each other, as we had predicted from ethnobotanical observation. Rather, the landraces grown in upland soils (ADE and Oxisols/Ultisols) are more related to each other and genetically differentiated from floodplain landraces. For landraces cultivated in the floodplain and in ADE, the selection for convergent adaptive traits appears to be associated with the similar ecological adaptations to nutrient-rich soils and short periods for rapid growth and yield before stress. The stresses are quite different, however: floods in the floodplain and enhanced weed pressure in ADE. Nonetheless, they yield convergent adaptive results.

If the similar ecological adaptations observed for ADE and floodplain landraces are outcomes of adaptive convergence of traits directed by farmer's selection, what drives that selection? On ADE, farmer selection for fast maturing landraces coupled with a reduction in fallow periods is likely to be driven by population pressure and permitted by ADE's high nutrient availability. When ADE is cultivated at Barro Alto and Água Azul, where population pressure exerts more of an influence, the most intensive swidden-fallow systems were found (Text S1). These intensive systems were associated with a reduction in the diversity of landraces in general, suggesting that when farmers reduce fallow lengths they may select only the fastest maturing landrace that is widely available for planting. In the floodplain, selection for fast maturing landraces is certainly a strategy to cope with the high seasonality of the floodplain determined by the flood pulse. Hence two different swidden-fallow systems have developed as convergent adaptations to intensification in nutrient-rich anthropogenic and floodplain soils. This intensification may be driven by population pressure (*terra firme*) and the flood pulse (floodplain); alternatively, owing to their greater fertility, it may be that the floodplain and ADE are the only places that offer the opportunity to produce food and/or money in a shorter time period – and this could also be a driver for the selection of these common traits.

Our findings broaden our understanding of the diversity of bitter manioc swidden-fallow systems in contemporary Amazonia, and allow some ethnographic projection. Firstly, the intensive short-fallow shifting cultivation in the floodplain and on bluffs with ADE that we reported along the middle Madeira are clearly modern analogs of Denevan's [17], [18] hypothesis that pre-conquest food production systems were much more intensive than the extensive long-fallow shifting cultivation common today, which he argues is a post-conquest adaptation. Secondly, Arroyo-Kalin [32] recently hypothesized that during the pre-Columbian period manioc was selected for high toxicity in swiddens in the infertile agricultural hinterland further from settlements, but farmers were selecting for lower toxicity in fertile ADE soils forming in and around homegardens. The phenomenon of farmer selection being driven by landrace agronomic performance in different soils would support the possibility that farmer selection in the pre-Columbian period for planting in different environments was a key driver in the emergence of “bitter” and “sweet” manioc and the continuum of toxicity that underlie them. Given the importance of bitter manioc cultivation to our understanding of pre-Columbian and contemporary populations in Central Amazonia, we hope that this study will encourage further research into the under-investigated area of intensified bitter manioc cultivation in fertile soils.

## Materials and Methods

### Study area and sampling design

We focus on the middle Madeira River, Amazonas State, Brazil (Figure 1), where traditional farmers plant their staple crop, bitter manioc, in infertile Oxisols and Ultisols of the *terra firme*, and in fertile ADE and floodplain soils. Amazonian Dark Earths are easily recognizable by their very dark brown or black coloring, high fertility, and pottery shards. The other *terra firme* soils were classified in the field according to local ethnopedological knowledge and physical properties (color, presence of potsherds, etc.). It was found that the local ethnopedological category “*barro*” (clay), recognized by its red/yellow coloring and high clay content in the A horizon, is broadly coterminous with Oxisols, while the local category “*areia*” (sand), recognized by dark brown, grey or black coloring and highly friable and “sandy” (although probably pseudosands) A horizon is broadly coterminous with Ultisols [26]. Along the Madeira River, farmers recognize three major zones in the floodplain: i) the highly fertile sides of the main channel, locally known as the *vazante*; ii) the high floodplain, known as the *restinga*; iii) and the back-swamp area, known as the *cacaia* (Figure 5). These terms are local and identify fewer categories than Denevan [68] observed along the floodplain of the Ucayali River, in eastern Peru, between the towns of Pucallpa and Panaillo.

Both the collection of plant material and the interviews took place after prior informed consent was obtained at each community. Anthropological fieldwork was authorized by a scientific expedition (EXC 022/05) granted by the Brazilian National Research Council (CNPq). Four *terra firme* localities (the second and third comprised of several communities) were selected for semi-structured quantitative interviews on bitter manioc cultivation on ADE, Oxisols and Ultisols: Barro Alto, Barreira do Capanã/Boa Vista, the Água Azul Coast, and Vista Alegre (Figure 1). These localities were selected because they had the greatest numbers of farmers cultivating this crop on ADE [83]. All farmers cultivating bitter manioc on ADE in these localities at the time of research were interviewed. We selected an equivalent number of families farming Oxisols and Ultisols using the snowball method whereby new families were enrolled through those who had already been interviewed ([84]:184–85). Two floodplain localities, each comprising several communities, were selected. Upstream from Manicoré families resident in the floodplain communities Fortaleza and Pau Queimado, and a few families at Água Azul and Monte Sião who had fields in the floodplain were selected. Downstream from Manicoré are the communities Verdum, Amparo and Delícia. These communities are among the longest established floodplain communities on the middle Madeira River. All farmers present at these floodplain communities during visits were interviewed. Key individuals who maintained the highest number of bitter manioc landraces were identified through open-interviews with different community members. In order to minimize gender/age biases, and to verify information from multiple sources, interviews were conducted in the household, normally with all of the family present, as family members would often intervene to correct the orator or provide additional information.

### Participant observation and open interviews

JAF conducted extensive participant observation in all communities of the study area prior to and during subsequent quantitative work (from September 2006 to March 2008), in order to build rapport and open up areas of interest that cannot be gleaned from close reading of the literature [85]. This entailed engaging in daily activities, including planting, harvesting and processing manioc, and conducting open, unstructured interviews with local people. The advantage of this type of initial qualitative approach is that it places no limitations on and is non-reductive in relation to reality. This open-ended approach led to the generation of the hypothesis that this paper addresses (that there are different adaptive manioc systems in different soils) and facilitated the discovery of the local theory of weakness and strength. The disadvantage of such an unstructured qualitative approach is that it cannot test the hypotheses it generates, and this is why we generated various novel quantitative indicators to measure aspects of bitter manioc cultivation systems from which adaptability could be inferred (planting behaviour, perceptions of landrace performance in different soils, yields and fallow lengths).

### Ethnobotanical Data, Indices and Analyses

In total, we conducted 249 semi-structured interviews at the six localities between May 2007 and March 2008. Quantitative ethnobotanical data were gathered on a) the area occupied by each manioc landrace (landrace area – *la* – see below) in fields on four soil types (ADE, Ultisols, Oxisols and Floodplain), b) perceptions of relative performance (performance ranking index – *pri* – see below) of the same landraces in the three different *terra firme* soils, c) fallow lengths, and d) incorporation of seedlings. In order to test if fields on different soils exhibit different compositions of landraces, data on *la* were ordered through a Non-Metric Multidimensional Scaling (NMDS) based on the Bray-Curtis dissimilarity, followed by non-parametric multivariate analyses of variance (MANOVA; [86]). Non-parametric MANOVAs were performed for the general model (i.e., including all soil types simultaneously) and also for the six possible pairwise comparisons between the soil types. Critical p-values were adjusted by the Holm correction for multiple comparisons [87]. The relationship between *la* and *pri* was investigated using simple correlations. Composite soil samples were collected in each field and the results from chemical analysis were used for an *a posteriori* classification of soil types [26].

### Landrace Area Index and Fallow Lengths

In order to identify the different landraces cultivated and quantify their abundance in the fields, we relied on the local nomenclature for the landraces and on numerical estimates provided by each farmer, using a local unit commonly used for quantifying manioc stem cuttings. In general, farmers along the middle Madeira recall the amount of each landrace they plant in terms of the number of bundles (“*feixes*”) of each landrace they planted in their fields. We asked each farmer (a) which landraces he/she cultivated, (b) how many bundles of each landrace he had planted in his/her current manioc field and (c) how big was his/her field. Using this information we calculated the area occupied by each landrace in each field (*la*) through the formula:
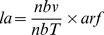
where *la* is the *landrace area* index, *nbv* is the number of bundles of a given landrace planted in a given field, *nbT* is the total number of bundles planted in that field, and *arf* is the area of the field. Since we did not observe differences in the spacing used to plant the landraces, we regard the area occupied by each landrace (*la*) as a good estimator of the abundance of each landrace in the field. When all landraces are taken into account, we interpret this data as the varietal composition of the fields. We collected data on fallow lengths by asking each farmer how old the fallow was that he/she had cleared in order to establish the field. Data for the calculation of *la* and on fallow lengths were collected with 249 farmers at all six localities, of which 190 farmers on the *terra firme* and 59 on the floodplain.

### Performance Ranking Index and seedling management

During participant observation it emerged that farmers claimed that certain landraces performed better in particular soils [83]. In order to quantify this, we asked all 190 *terra firme* farmers (a) to name all landraces that they cultivated in each type of soil and then (b) to rank the landraces mentioned according to their performance in that specific soil. Based on Sutrop's “Cognitive Salience Index” [88], we combined data on the frequency with which a given landrace was cited, its mean position in the performance ranking and calculated a Performance Ranking Index (*pri*) of each landrace in each type of soil, using the formula:
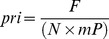
where *pri* is the Performance Ranking index, *F* is the frequency of citations of the landrace, *N* is the number of farmers interviewed who cultivate in this specific type of soil, and *mP* is the mean position of the landrace in the rankings.

Participant observation also revealed that some farmers purposefully separate cuttings from volunteer seedlings for later planting, others simply harvest them together with other landraces (resulting in their random incorporation into pre-existing landraces), and others purposefully exclude volunteers [83]. Once these three categories were established, the incorporation of seedlings was measured by asking 114 farming families if they: a) *separated* seedlings, that is took cuttings from seedlings and planted them apart to see how they did; b) *randomly* incorporated seedlings, where they do plant cuttings of seedlings volunteers, but randomly mixed up with the other landraces; or c) *removed* them, cutting the volunteers out as weeds when they appeared in the field.

### Farmer landrace yield estimates

Thirty one farmers were selected to record the production of different bitter manioc landraces in mono-varietal patches within 43 fields on the four different soil types. They were selected on the basis of literacy, their expressed interest in participating in the study (i.e., willingness to commit to recording production), and, crucially, the fact that they had mono-varietal patches within manioc fields during the study period. Bitter manioc fields are harvested bit by bit depending on labor availability and demand for manioc flour for subsistence consumption and sale. Amazonian farmers also do not tend to have accurate scales. They take a great interest in the amount of manioc flour produced, however, since it is equivalent to, as one woman put it, their “daily bread” (e.g., carbohydrate staple) and additionally an important product for sale in markets. In order to measure production therefore we asked farmers to record how many sacks of manioc flour (each sack is 50 kg) they produced from each mono-varietal-landrace patch. Fields were visited and we did not record significant differences in spacing, so we consider them to be all planted at similar density (around 1 meter between plants – c. 5000 plants per 0.5 ha). The size of mono-varietal patches varied (mean 0.286 ha±0.123 SD), so we corrected each one up to 0.5 ha in order to make the data commensurate and comparable using ANOVA (Table 1).

### Collection of genetic data

The collection of plant material for genetic analyses was carried out in 2009. Authorization for interviewing farmers was obtained from *Instituto Nacional de Pesquisas da Amazônia*'s Committee for Research Ethics (protocol 235/09) and our collecting was registered in the System for Authorization and Information on Biodiversity, coordinated by the Chico Mendes Institute for Biodiversity, of the Ministry of the Environment (number of register: 10020-5). No proprietary traditional knowledge was accessed, allowing us to meet Resolution 21 requirements for basic research that does not require authorization from Brazil's Council for Genetic Patrimony (CGEN in the Brazilian acronym), which was consulted before field work.

To investigate how the genetic diversity of bitter manioc was organized in different environments of cultivation we collected leaf samples of each landrace present in each of a total of 48 swiddens in all the localities except Vista Alegre: 17 in ADE soils, 14 in floodplain soils and 17 in Oxisols/Utilsols. A total of 184 individuals were sampled, representing 43 different bitter manioc landraces. The genetic variation of landraces was accessed using ten microsatellite loci [89], [90], which are short sequences of repetitive DNA. Seven of them (GA21, GA126, GA131, GA134, GA136, GA140, GAGG5) were described by Chavarriaga-Aguirre *et al.* [89] and three (SSRY13, SSRY89, SSRY 164) by Mba *et al.* [90]. Each microsatellite locus refers to a unique genomic region and the genetic variation results from differences in the number of repetitive units among individuals.

To investigate the extent of genetic variation within landraces and the genetic identity of landraces with the same name but grown in different soil types, some of the most commonly cultivated bitter manioc landraces in the region (*Pirarucu Branco*, *Tartaruga*, *Arroz*, *Jabuti* and *Pirarucu Amarelo*) had 20 or 30 individuals sampled (Dataset S2). To evaluate the dispersion of bitter manioc swiddens along the genetic diversity revealed by microsatellites, a Principal Coordinate Analysis (PCoA), based on Euclidian distances, was carried out with GenAlEx v.6.4 [91]. Among the parameters used to describe intra-varietal genetic diversity, we estimated the observed (*H_O_*) and expected (*H_E_*) heterozygotes with GenAlEx v.6.4, while the number of multi-locus genotypes (MLGs) was analysed with GenClone v.2.0 [92]. *H_O_* varies from 0 to 1, and corresponds to the probability that a given microsatellite locus has two different numbers of repetitions in a given individual. *H_E_* also varies from 0 to 1, and corresponds to the proportion of heterozygote individuals that were expected under the Hardy-Weinberg Equilibrium based on the number of different microsatellite forms found for a given landrace. The MLGs describe the number of different microsatellite combinations present in a given group of individuals (in this case, the landraces). The detailed sampling strategy, material and methods and other genetic results are described elsewhere [76], [77].

## Supporting Information

Dataset S1 **Landrace area and performance ranking index.**
(XLSX)

Dataset S2 **Intra-varietal genotypes and soil differentiation genotypes.**
(XLSX)

Text S1 **Manioc cultivation at the community level.**
(DOCX)
