# Supplementary material for: Convergent Adaptations: Bitter Manioc Cultivation Systems in Fertile Anthropogenic Dark Earths and Floodplain Soils in Central Amazonia
Source: PLoS One. 2012 Aug 29;7(8):e43636. doi: 10.1371/journal.pone.0043636 (PMC3430692; doi:10.1371/journal.pone.0043636)
Supplement: Text S1 — Manioc cultivation at the community level. (DOCX) [file pone.0043636.s003.docx]

**Supplementary Text S1: Manioc cultivation at the community level**

**Barro Alto.** This community is situated on a high *terra firme* bluff at a bend in the Manicoré River (a tributary of the Madeira River, Figure 1), which has much less river traffic than the main channel of the Madeira, and this community therefore has restricted market access compared to the communities located on the main channel. Bitter manioc cultivation is the principal livelihood activity of most inhabitants and the community is a local center for the production of this crop. Other livelihood activities include fishing and seasonal gathering of forest products, such as Brazil nut.

Barro Alto is one of the largest rural communities in the municipality of Manicoré, with around 600 residents, and also has a high population density: most houses are located within an area of less than two square kilometers. Forty years ago the community was comprised of only eight families with 62 people; these eight families have grown tenfold over the last four decades. This is an interesting anomaly in the general trend of rural-urban migration, which has seen an emptying out of rural Amazonia in recent decades [S1]. The community spans five landholdings: Bolívia, Barro Alto, Raimiro, Parintintin and Liberdade. Contrary to other communities, population density and pressure are high at Barro Alto. The majority of the population is crowded together on only three landholdings (Barro Alto, Raimiro and Parintintin; over 500 people in an area of 1500 x 500 meters). Liberdade is a large landholding with a single owner, and few families are allowed to cultivate there. This circumscribes the land available for occupation and cultivation, reducing the availability of land near the village. The large and growing population, with land tenure limiting access to land, has had the effect of reducing fallow periods on the ADE and Ultisols closest to the village, that are desirable because of ease of access, but especially on ADE, which can withstand more intensive cultivation.

Twelve landraces of bitter manioc are cultivated in this community. Bitter manioc is cultivated intensively, and the cropping cycles are significantly shorter on ADE (N = 36; fallow age: 5.3 ± 8.5) and Ultisols (N = 25; 10.5 ± 9.8) than on Oxisols (N = 33; 31.5 ± 18.7) (ANOVA F = 35.9; Tukey's post-hoc test: p < 0.001). Farmers also claimed that fields on ADE are fallowed more to relieve weed pressure rather than to restore fertility, which supports de Rouw’s [S2] hypothesis, for fertile soils at least. Among the nine landraces cultivated in ADE, *Tartaruga* is the most prominent: it was cultivated on 97% (35) of the fields on ADE, occupying 65.3% (13.5 ha) of the area cultivated on this soil. Farmers explained that *Tartaruga* is a fast maturing landrace with most people beginning to harvest only six months after planting. Genetic data showed that *Tartaruga* at Barro Alto is unrelated to the floodplain landrace of the same name planted in the floodplain at Verdum, Amparo and Delicía communities (see [S3,S4] for full analysis). There is a high degree of genetic divergence between the two, and the landrace *Tartaruga* grown in ADE and Oxisols had the preponderant MLG in common, which was different from the preponderant MLG found for *Tartaruga* from the floodplain (Dataset S2).

Ultisol cultivation tends to be somewhere between ADE and Oxisols, both in terms of the mix of landraces and the length of fallowing. In total, eight landraces are cultivated in Ultisols at Barro Alto. *Tartaruga* is also cultivated in this soil (it is present in 80% (21) of the fields and occupies 29.2 %; 5.2 ha of the area), but the most prominent landrace in Ultisols in Barro Alto is *Roxinha Amarela*, cultivated in 80% (20) of the fields and occupying 56.5 % of the area, or 10 ha. Ultisol cultivation is reasonably intensive, because these soils are located close to the village.

Oxisol cultivation is the most extensive, with the highest fallow age among all three types of soil (31.5 ± 18.7 years). Almost half (48.5%, 16) of the fields in this soil had been opened from mature forest, this could be a result of high population pressure, which causes people to open older fallow and mature forest owing to the lack of available areas with younger fallow. All the 12 landraces cultivated at Barro Alto were found in Oxisols, but the most predominant landraces were *Roxinha Amarela* (present in 100%; (33) of the fields and occupying 42,1% (16 ha) of the area) and *Arroz* (present in 75% (25) of the fields and occupying 21.7% (8.25 ha) of the area). Farmers mentioned that these landraces are harvested later and are slower-maturing than *Tartaruga*.

**Barreira do Capanã and Boa Vista.** At these communities the main livelihood activities are bitter manioc cultivation, fishing, hunting, fruit production in homegardens, rubber and Brazil nut extraction, with some timber extraction. The community is located behind a seasonal lake, which dries up from July-October, restricting market access during these months.

These communities have a lower population density than Barro Alto, with families (206 people in total) distributed in a series of 15 historic landholdings strung along a 6 km stretch of bluff. Each landholding is 1-4 ha or more in size, and is normally inhabited by between 10-20 individuals. Bitter manioc is planted in a large extension of ADE (some 50 ha in total) that runs along the bluff for 3 km, and in the Oxisols and Ultisols behind it. Farmers cultivate 19 landraces of the crop. There was no significant difference in fallow length between the three types of soil, although there is a tendency for fallow periods to be shorter in ADE (N = 17; 10.9 ± 6.5 years) when compared to Ultisols (N = 12; 16.3 ± 5 years) and Oxisols (N = 11; 14.3 ± 6.5 years). Fallow periods are longer in ADE in this locality compared to Barro Alto; possibly an outcome of lower population pressure. Fourteen landraces are cultivated on ADE, with the most predominant being *Pirarucu Branco* (present in 58.9% (10) of the fields and occupying 27.7 % (2.69 ha) of the area cultivated on ADE), *Tartaruga* (52.9 % (9) of the fields and 17.9 % (1.74 ha) of the area) and *Arroz* (52.9 % (9) of the fields and 12. 8 % (1.24 ha) of the area). A landrace named *Pirarucu Branco* is also planted in the upstream floodplain area (community Pau Queimado), but strong genetic differentiation was found between the landrace *Pirarucu Branco* planted at Barreira do Capanã and the landrace with the same name planted in the floodplain in Pau Queimado (Table 3; see [S3,S4]). However, it was shown that the preponderant MLG of the landrace *Pirarucu Branco* was present in some individuals from the *Arroz* landrace planted in ADE at Barreira do Capanã (Table 3). This may explain why farmers at this locality claim *Arroz* performs well in ADE: *Pirarucu Branco* and *Arroz*, at least in this community, may have related genetic features.

On Ultisols, eight landraces are cultivated, the predominant being *Jabuti* (present in 75 % (9) fields and occupying 29.7 % (3.27 ha) of the area) and *Roxinha* (66.7% (8) of the fields and 25 % (2.7 ha) of the area). On Oxisols, 13 landraces are cultivated, the predominant being Jabuti (81.8 % (9) of the fields and 38.5 % (3.85 ha) of the area) and *Arroz* (72.7% (8) of the fields and 22.7 % of the area).

Owing to lower population pressure, cultivation of ADE is less intensive at Barreira do Capanã than at Barro Alto, and the tendency for fields to be cleared from young fallow in ADE is therefore less pronounced. Oral histories indicate that the population has fallen over the last 150 years, which explains this. Consequently, fallow lengths are much more similar across the three soil types, with the exception of a few more intensively cultivated ADE fields. The low population pressure is also reflected in the much lower number of fields opened from mature forests in this locality compared to Barro Alto (two fields out of 40 (5 %) on Barreira do Capanã and 18 fields out of 94 (19,1 %) on Barro Alto).

**The Água Azul Coast.** This area of *terra firme* bluffs encompassing the communities of Água Azul, Monte Orebe and Monte Sião on the North Bank of the Madeira River. A principal livelihood activity in this region for those with tenure is cultivating plantain and banana in the *restinga* on the opposite side of the river. Watermelon, maize, beans and bitter manioc are also cultivated in the *vazante* floodplain in front of the community. Bitter manioc cultivation, homegarden fruit production on the *terra firme*, and the gathering of forest products are also important. These communities have good market access, owing to their proximity to the main channel.

Água Azul is a relatively densely populated community, with 207 people occupying a 2 km stretch of bluffs, divided into 15 small (0.5-1.5 ha) family landholdings, each occupied by between 10 and 20 people, while Monte Orebe and Monte Sião are sparsely populated, with 60 and 35 residents, respectively, occupying several landholdings (1-3 ha) located along 6 km of bluffs. Until 30 years ago, residents of these communities (and many of those now living at Vista Alegre) inhabited a high floodplain in front of where they now live, but this was washed away as the main channel of the Madeira shifted.

The main ADE site at the Água Azul community has been very intensively cultivated under a short-fallow swidden system by one extended family for the past thirty years. Lacking sufficient land for long fallowing, because all land close to the community is either fallow or owned by other families, the family developed an intensive system reminiscent of that at Barro Alto, with a floodplain landrace (*Pirarucu Amarelo*) as its focus, and short fallows (5.9 ± 6.7 years). Although there is no significant difference in fallow length between the different types of soil, fallow periods tend to be shorter on ADE than on Ultisols (11.1 ± 6.2 years) and Oxisols (8.7 ± 4.8 years). Farmers at Água Azul cultivate nine landraces in total, eight of which are present in ADE. *Tartaruga* was the most predominant landrace on ADE (present in 62.5 % (5) of the fields and occupying 36.7 % (2.3 ha) of the area), followed by *Pirarucu Amarelo* (50 % (4) of the fields and 17.4 % (1.1 ha) of the area) and *Pirarucu Branco* (37.5 % (3) of the fields and 16.1 % (1 ha) of the area). Genetic results determined that *Pirarucu Amarelo* that is planted in the floodplain at Água Azul (and possibly at Pau Queimado and Fortaleza) is identical to the landrace of the same name planted at the *terra firme* in Água Azul (Table 3). The ADE site at Monte Sião is a large site that was abandoned after a land dispute but has recently been re-occupied by two families. They plant *Pirarucu Branco* and *Tartaruga* in their ADE, similar to other ADE sites. There are two other smaller patches of ADE, one on the bluff overlooking Igarapé Água Azul and another behind the village at Monte Orebe.

The Ultisol and Oxisol fields, while located in older fallow than ADE, are still located in relatively young fallows (Oxisols 8.7 ± 4.8 years; Ultisols 11.1 ± 6.2 years), owing to population pressure. The primary forest directly inland is now distant and land-tenure restricts access to other areas of easier access. Eight and six landraces are cultivated in Ultisols and Oxisols, respectively, but in both soils the predominant landraces are *Arroz* and *Jabuti*. In Ultisols, *Arroz* occurs in all fields sampled (8) and occupies 42 % (2.73 ha) of the area cultivated in this soil, while *Jabuti* occurs in 50 % (4) of the fields and occupies 23 % (1.5 ha) of the area. In Oxisols, *Arroz* occurs in 62.5 % (5) of the fields and occupies 17.9 % (1.3 ha) of the area, while Jabuti occurs in all fields sampled (8) occupying 53.1 % (3.85 ha) of the area cultivated in this soil.

In the Água Azul coast we see a pattern that is broadly similar to Barro Alto and Barreira do Capanã / Boa Vista. There is a significantly greater presence of the most popular slow-maturing landraces, *Jabuti* and *Arroz*, in Oxisols and Ultisols when compared to ADE. *Tartaruga* and *Pirarucu Amarelo* are the most popular in ADE, but the latter is also planted in other soils. Fast maturing *Pirarucu Branco* and *Tartaruga* have recently arrived and are becoming popular on the ADE sites at Monte Sião and Monte Orebe. *Pirarucu Amarelo* is planted in Oxisols, Ultisols and ADE because it yields very yellow manioc flour.

**Vista Alegre.** At this community, cultivation of plantain, banana and watermelon in the high floodplain directly in front of houses and on the other side of the river are important livelihood activities. Vista Alegre also has good market access owing to its proximity to the main river channel, although access is hampered during the dry season by the drying up of watercourses in the floodplain directly in front of the community. Bitter manioc cultivation takes place on the *terra firme* behind the community.

Vista Alegre is a relatively large community of 294 people occupying 3 km of bluffs, divided into eight large landholdings, each inhabited by several family clusters, located behind a floodplain forest along the main channel of the Madeira River. At Vista Alegre there is relatively high varietal diversity; 21 landraces were recorded in total. 18 of these were present in ADE, with the predominant landrace in ADE being *Roxinha Branca* (present in 60 % (6) of the fields, occupying 22.6 % (1.4 ha) of the area), followed by *Amarelinha* (20 % (2) of the fields and 13.1 % (0.82 ha) of the area). Oral histories recall that this landrace was brought from the floodplain to the *terra firme,* when today’s inhabitants migrated from there. Fallow periods are shorter in ADE (4.6 ± 4.2 years) than on Ultisols (16.4 ± 4.7 years) and Oxisols (11.3 ± 3.7 years; ANOVA F = 19.73; p < 0.001; Tukey's post-hoc test: p = 0.023 (Oxisol vs. Ultisol), p < 0.001 (Ultisol vs. ADE), p = 0.002 (Oxisol vs. ADE)). The combination of more faster maturing manioc landraces and shorter fallows in ADE means that -- as at Barro Alto -- there are more intensive systems in these soils.

The predominant landrace in Oxisols and Ultisols is *Jabuti*, present in 91.6 % (11) and 100 % (10) of the fields, and occupying 52.7 % (5.5 ha) and 72.3 % (6.7 ha) of the area cultivated in these soils, respectively. In Oxisols, *Arroz* is also important, occurring in 41.7 % (5) of the fields and occupying 14.3 % (1.5 ha) of the area. Longer fallows in Oxisols and Ultisols demonstrate that the bitter manioc cultivation is more extensive in these types of soil.

**The Floodplain at Água Azul, Pau Queimado and Fortaleza.** This floodplain area upstream from Manicoré consists of families from three neighboring localities: the community of Água Azul on the *terra firme*, where manioc is planted in the adjacent floodplain *vazante*, and the floodplain communities of Fortaleza (pop. 90) and Pau Queimado (pop. 130), where manioc is planted in the *cacaia* and *restinga*. At Fortaleza and Pau Queimado, the floodplain is too narrow (only 250 m wide) to permit large-scale cultivation of cacao and plantain; consequently bitter manioc cultivation is an important livelihood activity. The floodplain is divided into small family landholdings, 0.25-0.5 ha in size. There are a total of 13 landraces cultivated at these three communities, 11 at Fortaleza, 6 at *Pau Queimado*, and 4 at Água Azul. The predominant landraces in these communities are *Pirarucu Amarelo* (present in 37.5 % (9) of the fields and occupying 22.5 % (2.9 ha) of the area), *Olho Roxo* (37.5 % (9) of the fields and 17.5 % (2.3 ha) of the area), *Mãe Joana* (50 % (12) of the fields and 15.3 % (2 ha) of the area) and *Juvenal* (50 % (12) of the fields and 14.8 % (1.9 ha) of the area). The former two are, according to farmers, the quickest to mature, and are planted in lower floodplain zones, while the latter two are slower maturing, and planted in higher zones. Indeed, the fastest maturing landrace, *Pirarucu Amarelo*, was most commonly planted in the lowest area of the floodplain: the *vazante* at community Água Azul. Most of the landraces planted in the floodplain are different from those planted in the *terra firme*, with a few exceptions. *Pirarucu Amarelo* - widely planted on the *terra firme* - is the most predominant landrace in the Água Azul floodplain. *Jabuti* and *Aruari*, other *terra firme* landraces, are also planted in the floodplain, albeit in small quantities. The social history of some landraces shows considerable mobility, and some may even move from the floodplain onto the *terra firme*, and then back into the floodplain. This is the case with the *Glaí* landrace. It was taken from a floodplain community called Pandegal by a woman named Glaí from Vista Alegre. The landrace became established in Vista Alegre, before moving along the *terra firme* to Barreira do Capanã. It then moved back into the floodplain at the Fortaleza community, becoming *Glaí Branco* (white) and *Glaí Amarelo* (yellow) in the process.

Interestingly, genetic analyses demonstrated that *Pirarucu Branco* planted in the floodplain at Pau Queimado had the same preponderant MLG as *Tartaruga* from the *terra firme* at Barro Alto (from ADE and Oxisol), but a different preponderant MLG from *Pirarucu Branco* that is planted in the *terra firme* [S3-S4]. We believe that the case of *Pirarucu Branco* could be due to a man from the Community of Boa Vista, who claimed to have been the first to bring *Tartaruga* from the Manicoré River to Boa Vista, where it spread to Barreira do Capanã and to the Água Azul region. When it moved into the floodplain, genotype-environment interactions may have produced a different morphotype, which farmers associated with those that they recognize as *Pirarucu Branco*, hence the renaming [S3].

**The Floodplain at Delícia, Amparo and Verdum.** The communities Delícia (pop. 100), Amparo (pop. 80) and Verdum (pop. 171) are located along an 8 km stretch of high floodplain in front of the Lake Genipapo. On the floodplain of Lake Genipapo, farmers in all three communities reside on the high floodplain, which is more than 750 m wide in some places. Because of this larger expanse of available floodplain land, landholdings are relatively large, ranging from 0.5 ha to 3 ha in size. The most important livelihood activity is the cultivation of plantain and cacao in the high floodplain. Farmers also plant manioc in the *cacaia* and on the *restinga*. There are a total of 14 manioc landraces cultivated at these communities, 12 at Delícia, 7 at Amparo, and 5 at Verdum. The predominant landraces planted at these communities are *Tartaruga* (present in 91.4 % (32) of the fields and occupying 33.1 % (8 ha) of the area), *Curuçá* (77.1 % (27) of the fields and 22.9 % (5.5 ha) of the area), *Sempre Serve* (74.3 % (26) of the fields and 22.9 % (5.5 ha) of the area) and *Abidãozinho* (25.7 % (9) of the fields and 5.1 % (1.25 ha) of the area). The first two of these are faster maturing and tend to be planted in lower areas of the *cacaia* and on the *restinga*. The third and fourth tend to be planted in higher areas of the *cacaia* and on the *restinga*. As previously discussed, it was demonstrated that the *Tartaruga* landrace from the *terra firme* and that cultivated in the floodplain are genetically distinct, although we observed in the field that they are morphologically similar, which could explain the shared name. The following quote demonstrates how local farmers recognize that there are morphologically similar but agronomically distinct landraces with the same name on the *terra firme* and in the floodplain:

“There is *Tartaruga* of the floodplain, and *Tartaruga* of the *terra firme*. Both of them are old here. My father planted both. The plant of both landraces is identical. Bright green leaves, white stem and white *talo* (petiole). The tuber is identical in that it has a very thin skin. What is different is that the floodplain landrace has a whiter tuber, and yields well in the floodplain and in *terra preta* (ADE). In the clay (Oxisols) it doesn’t yield well. The *terra firme* *Tartaruga* yields in the floodplain, but takes longer to mature. It yields well in *terra preta* and clay. The floodplain landrace matures in a year. The *terra firme* landrace takes longer to mature, around 2 years.”

Luci Leli Carlos Pinheiro, Community Delícia

S1. Parry L, Day B, Amaral S, Peres CA (2010) Drivers of rural exodus from Amazonian headwaters. Population and Environment 32: 137-176.

S2. de Rouw A (1995) The fallow period as a weed-break in shifting cultivation (tropical wet forests). Agriculture, Ecosystems and Environment: 31-4354.

S3. Alves Pereira A (2011) Dinâmica evolutiva de mandioca (Manihot esculenta Crantz) em três tipos de solos manejados por Caboclos na região do Médio Rio Madiera, Amazonas. Manaus, Brazil: Instituto Nacional de Pesquisas da Amazônia. 87 p.

S4. Alves-Pereira A, Peroni N, Abreu AG, Gribel R, Clement CR (2012) Genetic structure of traditional varieties of bitter manioc in three soils in Central Amazonia. Genetica 139: 1259-1271.
